# Fosfomycin-resistant *Escherichia coli*: a FosA10 case in Italy

**DOI:** 10.1093/jacamr/dlaf052

**Published:** 2025-04-09

**Authors:** Vittoria Mattioni Marchetti, Ilaria Petrizzi, Irene Venturelli, Tiziana Cassetti, Marianna Meschiari, Roberta Migliavacca, Ibrahim Bitar

**Affiliations:** S.C.C.D.P. Department, Microbiology and Clinical Microbiology Unit, University of Pavia, Pavia, Italy; S.C.C.D.P. Department, Microbiology and Clinical Microbiology Unit, University of Pavia, Pavia, Italy; Clinical Microbiology, AUSL Modena, 41125 Modena, Italy; Clinical Microbiology, AUSL Modena, 41125 Modena, Italy; Infectious Diseases Clinic, AOU Policlinico di Modena, Modena, Italy; S.C.C.D.P. Department, Microbiology and Clinical Microbiology Unit, University of Pavia, Pavia, Italy; I.R.C.C.S. Policlinico S. Matteo, SC Microbiology and Virology, Department of Diagnostic and Laboratory Medicine, Pavia, Italy; Department of Microbiology, Faculty of Medicine, University Hospital in Pilsen, Charles University, Pilsen, Czechia; Biomedical Center, Faculty of Medicine, Charles University, Pilsen, Czechia

## Abstract

**Background:**

FosA10-producing Enterobacterales have an extremely low incidence in Europe.

**Patients and methods:**

In March 2024, an 83-year-old woman, hospitalized in the Modena Province, developed an infection with fosfomycin-resistant *Escherichia coli*. The patient was treated with piperacillin/tazobactam and, after 10 days, the clinical picture was resolved. Fosfomycin MIC was evaluated with the reference agar dilution method and the production of FosA enzymes by phenotypic testing. Genomic characterization was assessed using long-read sequencing technology on the Sequel I platform.

**Results:**

An *E. coli* isolate (FO_2) was collected from both blood and urine samples and showed high-level resistance to fosfomycin (MIC > 128 mg/L). The resistance to fosfomycin was ascribed to the production of FosA-like enzymes by phenotypic testing. The genomic analysis pointed to a FosA10-producing *E. coli* ST69. The *fosA10* gene was carried by a highly conjugative IncB/O/K/Z plasmid that showed relevant similarities with other globally circulating plasmids.

**Conclusions:**

The acquisition of rare *fosA*-like genes in clinically relevant clones is concerning and the dissemination of FosA-producing *E. coli* should be continuously monitored.

## Introduction

Urinary tract infections are among the most common community-acquired and healthcare-associated bacterial infections, with a higher incidence in women than men,^1^ and uropathogenic *Escherichia coli* are responsible for the majority of uncomplicated and complicated urinary tract infections.^1^ Moreover, extraintestinal pathogenic *E. coli* of STs 69,^2^ 73,^2^ 95^2^ and 131^2^ are often associated with urinary tract infections and bloodstream infections.^2,3^ Fosfomycin is an ‘old’ antibiotic used as first-line treatment against urinary tract infections and recently reintroduced into clinical practice against MDR Enterobacterales species. Fosfomycin is a phosphonic acid derivative that binds the UDP-*N*-acetylglucosamine enolpyruvyl transferase enzyme, interfering with peptidoglycan production, which results in a bactericidal activity. The phenomenon of fosfomycin resistance is increasing worryingly, and the production of acquired hydrolysing enzymes (FosA-like) represents the most widespread mechanism. The FosA-like family includes glutathione S-transferase enzymes that inactivate fosfomycin by the addition of glutathione, and they are spread among clinically relevant bacteria such as *E. coli,*^4^  *Klebsiella pneumoniae*^4^ and *Salmonella enterica.*^4^ So far, 10 different variants of *fosA* (*fosA1–fosA10*) have been described in the literature, mostly located on plasmids in *E. coli*,^5–8^  *K. pneumoniae*^9^ and *S. enterica*^10^ isolated from human^5^ and livestock animals.^6–8^  *fosA3* is the most frequent gene found worldwide, while variants such as *fosA8* and *fosA10* are rare.^11^ In particular, *fosA10* was firstly described in 2020 by Huang and colleagues^12^ from a local broiler meat outlet in Pakistan, and subsequently in the Czech Republic from a rectal swab.^13^ In Europe, *fosA*-like genes are located on plasmids belonging to various incompatibility groups such as IncF,^14^ IncN,^14^ IncA/C,^14^ IncHI2^14^ and the IncX1 family.^14^ The spread of fosfomycin resistance in *E. coli* involves a large number of different STs, including high-risk clones such as ST131,^15^ ST69^16^ and ST648.^13^ ST69 is an extraintestinal pathogenic *E. coli* lineage that is globally involved in urinary tract infections from both the community^17^ and the hospital environment.^18^ Originally, *E. coli* ST69 was susceptible to almost all the antibiotic families, but the acquisition of *fosA*-like genes is affecting the use of fosfomycin in urinary tract infection treatment.^2,19^

The aim of the study was to characterize the genomic features of the first Italian case of plasmid-mediated *fosA10* in a clinical *E. coli* strain from Modena Hospital, Italy.

## Materials and methods

### Patient information

On 12 March 2024, an *E. coli* isolate (FO_2) was collected from a urine sample and, subsequently, from a blood culture of a feverish 83-year-old woman who was hospitalized for more than 30 days in a Medicine Unit of a hospital in the Modena Province. The patient was treated with piperacillin/tazobactam. Ten days later the control urine culture was negative and, 15 days after the isolation of the microorganism, given the resolution of the clinical picture, the patient was discharged. In the following months, no more culture tests were requested for the patient but only biohumoral control tests on an outpatient basis. The following investigations were conducted on FO_2 only.

### Identification of bacterial isolate, susceptibility determination and FosA enzyme detection

Species identification and susceptibility profile were carried out using the MicroScan AutoSCAN4 System (Beckman Coulter). Fosfomycin MIC was assessed via the EUCAST reference agar dilution method and interpreted according to EUCAST 2024 criteria (https://www.eucast.org/fileadmin/src/media/PDFs/EUCAST_files/Breakpoint_tables/v_14.0_Breakpoint_Tables.pdf), while the presence of acquired FosA enzyme was confirmed by using disc potentiation testing with sodium phosphonoformate (foscarnet), as reported elsewhere.^5^

### Long-read sequencing

For genomic characterization, genomic DNA was extracted using the NucleoSpin Microbial DNA kit (Macherey-Nagel, Duren, Germany) and sheared using the Hydropore - long on Megaruptor 2 (Diagenode). Microbial multiplexing library preparation was performed without size selection according to the manufacturer’s instructions. The multiplexed library was sequenced using long-read sequencing technology using the Sequel I platform (Pacific Biosciences, Menlo Park, CA, USA) for a 10 h movie run. Assembly was performed using the ‘Microbial Assembly’ pipeline offered by the SMRT Link v10.0. with the default settings (minimum seed coverage of 30×). Assembled sequences were annotated using the Rapid Annotation using Subsystems Technology (RAST) server^20^ and checked manually. The MLST profile was assigned according to the Achtman scheme on Enterobase v1.2.0,^21^ while plasmid MLST (pMLST) was investigated through pMLST (https://pubmlst.org/organisms/plasmid-mlst). Reconstruction of the resistome, plasmidome and virulome of the isolates was accomplished using ResFinder, PlasmidFinder and the Virulence Factors Database (VFDB) via ABRicate (github.com/tseemann/ABRicate). BRIG v.0.95 was used to produce figures of comparison of the circular plasmid sequences.^22^ Maximum-likelihood phylogeny using FastTree^23^ was constructed, comparing 88 available IncB/O/K/Z plasmid sequences retrieved from the plasmid database (PLSDB) (https://ccb-microbe.cs.uni-saarland.de/plsdb2025) against pFOS.

### Conjugation assay

The conjugal transfer of the *fosA10* gene was tested in liquid medium using the *E. coli* J53 (rifampicin resistant; RIF^r^) and *E. coli* J62 strains (streptomycin resistant; SM^r^) as recipients. Transconjugants were selected on MacConkey agar plates (Scharlab, SL, Barcelona, Spain) containing rifampicin (100 mg/L) or streptomycin (1000 mg/L) (Sigma–Aldrich, St. Louis, MO, USA), fosfomycin (64 mg/L) (Sigma–Aldrich) and glucose-6-phosphate (25 mg/L) (Roche). The presence of *fosA10* and the plasmid content in transconjugants were further confirmed by PCR and PCR replicon typing (PBRT 2.0 kit, Diatheva), respectively.^24^ Fosfomycin MIC for transconjugants was checked by the agar dilution method.

### Data availability

Nucleotide sequences for FO_2 have been uploaded in NCBI databases under the BioProject accession number PRJNA1186375.

## Results

FO_2 showed a fosfomycin resistance profile (MIC ≥ 64  mg/L; resistant) on MicroscanAutoSCAN4. The resistance to fosfomycin was confirmed by the agar dilution method (MIC > 128 mg/L) and the production of FosA enzyme was detected by the foscarnet test.

WGS of FO_2 (coverage 100×) revealed the presence of an *E. coli* ST69 O17/O44/O77:H18 of phylogroup D and harbouring the *fosA10* variant, together with genes involved in resistance to β-lactams (*bla*_TEM-1b_), macrolides [*mph*(A)], tetracycline [*tet*(B)] and trimethoprim (*dfrA14*). The *in silico* plasmid evaluation specified the presence of two plasmids only: a large multi-replicon plasmid (IncFII-FIA-FIB-IncY-Col156) of 208 572 bp and an IncB/O/K/Z plasmid of 82 187 bp. Concerning the virulence power, FO_2 showed a hybrid virulence content, containing both chromosomal and plasmid-related genes restricted to both uropathogenic and enteroaggregative *E. coli* (EAEC): the virulome included genes involved in adherence (*air*, *csgA*, *eilA*, *fdeC*, *fimH*, *iha*, *lpfA* and *papA*), metabolism (*chuA*, *fyuA*, *gad*, *irp2*, *iucC*, *iutA*, *sitA*, *terC*, *nlpl*), capsule (*kpsE*, *shiA*), toxicity (*sat*, *senB*, *hlyE*), invasion (*aslA*, *iss*, *traT*, *yehC* and *yehD*) and chemotaxis (*hra*).

**Figure 1.**
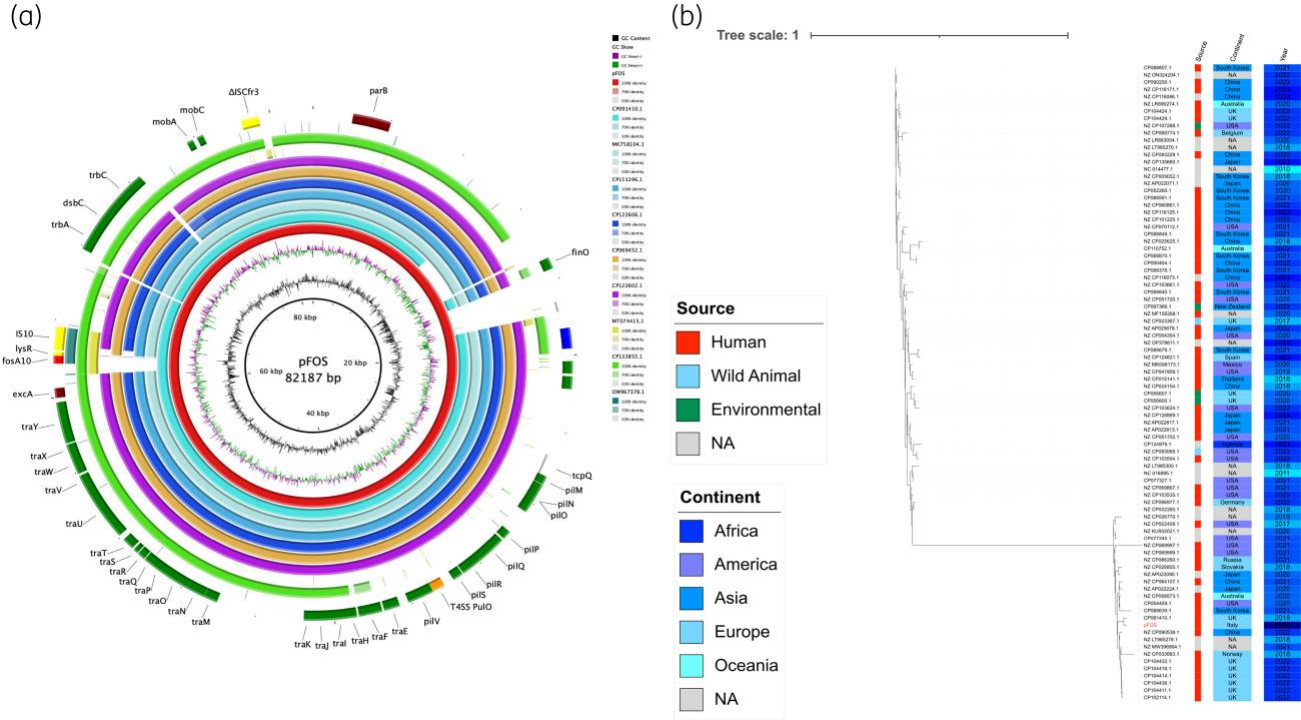
(a) BRIG circular map of pFOS against CP091410.1 (turquoise), MK758104.J (baby blue), CP151296.1 (sky blue), CP122606.1 (royal blue), CP069452.1 (orange), CP122602.1 (purple), MT074415.1 (yellow), CP133855.1 (green), OW967378.1 (chrome green). At the outer curved segments, blue, yellow, red, green, orange and brown refer to replication (repA), IS, AMR genes, conjugation system, T4SS and partitioning system locus. (b) Maximum-likelihood phylogenetic tree of pFOS and 88 available genomes of IncB/O/K/Z plasmids retrieved from PLSDB, using FastTree and pictured using iTOL v6.^25^ The alignment was performed with MAFFT v7.525 (https://mafft.cbrc.jp/alignment/software/source.html).

Based on both WGS and conjugational experiments, the *fosA10* variant was located on the IncB/O/K/Z plasmid (pFOS). *fosA10* was located on a 6082 bp cassette composed of the following: IS*10*-*LysR*-*fosA10*-DEAD BOX-hypothetical-*excA*. The same cassette is shared with plasmids from: ECO49406, a clinical *E. coli* collected in the Czech Republic in 2019; CP091410.1 (p542093), a clinical *E. coli* ST227 collected in the UK in 2018 and causing travellers’ diarrhoea; MT074415.1 (pHNPK9-Fos), an avian *E. coli* in China in 2020; CP133855.1 (ES4 plasmid), a clinical *E. coli* ST167 from the UK; and OW967378.1 (P4 plasmid) a clinical *Klebsiella oxytoca* collected in 2018 in Spain (Figure 1a). Interestingly, pFOS shared a high identity level with p542093, suggesting a possible connection route with the UK. pFOS was enriched with genomic loci involved in conjugational processes such as *tra* locus, *dsbC*, *trbAC*, *mobAC*, *pil* locus and the type IV secretion system (T4SS, *pulO*) (Figure 1a). Indeed, pFOS was highly transferable by conjugation in both *E. coli* J53 and J62, and all the obtained transconjugants harboured high-level fosfomycin resistance (MIC > 128 mg/L).

Phylogeny analysis of pFOS against 88 IncB/O/K/Z plasmids showed clustering with p542093 (CP091410.1). Moreover, pFOS and p542093 were included in a multi-branching cluster, showing a genomic relationship with plasmids detected in China, Norway and the UK since 2018 (Figure 1b). These data suggest a likely origin within the Chinese human field and a preferential transmission route from the UK environment.

## Discussion

Here we report the first entry of IncB/O/K/Z-harbouring *fosA10* in an Italian hospital setting.

Fosfomycin resistance represents a relevant threat, emerging globally and affecting the use of fosfomycin in severe infections with Enterobacterales. Comprehensive knowledge on this phenomenon is limited and challenging due to the scarcity of both rapid diagnostic approaches for fosfomycin MIC evaluation^11,26^ and specific surveillance programmes.^11,26^ In accordance with both EUCAST and CLSI, the agar dilution method remains the reference method for fosfomycin MIC evaluation,^11^ but it is a time-consuming and skill-demanding method. A time-saving and ready-to-use alternative is represented by the commercial agar dilution kit, but it is scarcely applied in routine practice due to its high cost.^26^ Alternatively, the disc diffusion method associated with foscarnet for *E. coli* strains can be useful in preliminary categorization (susceptible/resistant) and in evaluating the production of FosA enzymes.^26^ The use of a commercial agar dilution method for high-risk pathogens with suspected fosfomycin resistance should be implemented in routine practice, providing some clarity on the fosfomycin resistance phenomenon among Enterobacterales.^11,26^ Furthermore, MICs of fosfomycin should not only be investigated in cases of severe infections but also in cases of asymptomatic bacteriuria, in order to have an overview of the circulation of fosfomycin resistance traits within a hospital setting.

According to the literature, *E. coli* ST69 is a clinically relevant pandemic clone that is widespread among different hosts, causing urinary tract infections and exhibiting resistance to antibiotics.^27–29^  *E. coli* ST69 has acquired different antibiotic resistance traits, including carbapenemases.^30,31^ In fact, *E. coli* ST69 is globally recognized as vehicle of ESBLs, such as *bla*_CTX-M-55_, within livestock^32,33^ and environmental^32^ niches.

Despite *fosA10* having a low—and unknown—incidence, the presence of *fosA10* in pandemic clones represents a further threat to the use of antibiotic therapy in the clinical setting. Moreover, the occurrence of the *fosA10* gene in a highly conjugative and globally widespread IncB/O/K/Z plasmid highlights the significance of this funding and its speculative spread potential. In the case here reported, the resistance to fosfomycin did not present an obstacle to resolution of the clinical picture, due to the restricted resistome of the bacterial strain involved and the availability of other first-line therapeutic options. However, the scientific literature has being enriched with cases of the co-presence of fosfomycin and β-lactam resistance phenotypes, including carbapenems.^34–36^

In this study the cause of infection remains unknown. As a matter of fact, based on the available data, the patient has not come into contact with any risk sources, nor have similar cases occurred within the same hospital. However, no environmental sampling was carried out and a dedicated surveillance plan was not implemented. These gaps limit our study and the possibility of tracking the circulation of such fosfomycin resistance traits. These limitations point out the need of surveillance programmes for the circulation of *fosA*-like genes among Enterobacterales, not only for epidemiological purposes but even for filling the information gap, essential for facing and solving further similar cases.

The plasmid genomic analysis revealed the involvement of a IncB/O/K/Z plasmid (pFOS) in *fosA10* spread. IncB/O/K/Z is a versatile and highly conjugative plasmid group, implicated in the dissemination of *bla*_CTX-M-15_^37^ and carbapenemases.^38^ pFOS showed highly enriched conjugative systems, leading to an elevated transferability power. The plasmid phylogeny highlighted a plasmid backbone shared with other globally reported plasmids, showing the conservative nature of pFOS. Moreover, pFOS kept high-level identity with *fosA10*-carrying p542093, circulating in the UK. This evidence could lead to the hypothesis of a connection between the UK and Italy as a diffusion route of *fosA10*-harbouring IncB/O/K/Z plasmids. However, the lack of similar plasmids within European countries and the poor knowledge on the global epidemiology of horizontally transferable fosfomycin resistance traits make such speculation difficult to validate.

This study presents several limitations. Firstly, the investigation was carried out only on the infected patient without including any asymptomatic people who had come into contact; this does not allow us to have the real incidence and diffusion of this infection. Secondly, the poor molecular characterization at the plasmid level of these traits in Europe does not provide an adequate number of comparisons that can shed light on the diffusion route of *fosA10*.

In conclusion, the presence of the rare FosA10 in a healthcare setting, within a highly conjugative plasmid, could pose a challenge for microbiological diagnosis and surveillance. So far, the route of transmission of rare *fosA*-like genes, including *fosA10*, in Europe is unknown and requires further investigation.^11,26^ Despite the limitless number of rapid diagnostic approaches for fosfomycin MIC evaluation, more effort is necessary in implementing fosfomycin surveillance programmes, together with molecular approaches, to better understand the prevalence of *fosA*-like genes in Italy and to maintain the optimal use of this antibiotic.
